# Eating disorder symptomatology and substance use disorders: Prevalence and shared risk in a population based twin sample

**DOI:** 10.1002/eat.20856

**Published:** 2010-08-23

**Authors:** Jessica H Baker, Karen S Mitchell, Michael C Neale, Kenneth S Kendler

**Affiliations:** 1 Virginia Institute for Psychiatric and Behavioral Genetics, Department of Psychiatry, Medical College of Virginia of Virginia Commonwealth UniversityRichmond, Virginia; 2 Department of Psychology, Virginia Commonwealth UniversityRichmond, Virginia; 3 Women's Health Sciences Division, National Center for Post-traumatic Stress Disorder, Veteran's Affairs Boston Healthcare SystemBoston, Massachusetts (as of 8/31/2009); 4 Department of Human Genetics, Virginia Commonwealth UniversityRichmond, Virginia

**Keywords:** bulimia nervosa, anorexia nervosa, disordered eating, substance use disorders, comorbidity, prevalence, twin study, genetics

## Abstract

**Objective:**

Research shows a significant association between eating disorders (ED) and substance use disorders (SUD). The objective of this study is to examine the prevalence, chronology, and possibility of shared familial risk between SUD and ED symptomatology.

**Method:**

Subjects included 1,206 monozygotic and 877 dizygotic adult female twins. ED symptomatology included anorexia (AN) and bulimia nervosa (BN) diagnosis, symptoms associated with diagnostic criteria, and BN symptom count. SUD included alcohol, illicit drug, and caffeine abuse/dependence. Generalized estimated equation modeling was used to examine phenotypic associations, and Choleksy decompositions were used to delineate the contribution of genes and environment to comorbidity.

**Results:**

There were no significant differences between SUD prevalence in women with AN and BN. Women with BN reported BN preceded SUD development while the reverse was true for AN. Twin analyses showed possible familial overlap between BN symptomatology and all SUD examined.

**Discussion:**

Results suggest an important difference in the chronology of EDs and SUDs. Women with BN may be turning to substances to dampen bulimic urges. Women with AN may be engaging in substance use initially in an effort to lose weight. Results also suggest familial factors contribute to the comorbidity between BN and SUD. © 2010 by Wiley Periodicals, Inc. (Int J Eat Disord 2010;)

## Introduction

Rates of substance use and substance use disorders (abuse/dependence) are high among women with eating disorders (ED). This association is greatest in women with bulimia nervosa (BN) and anorexia nervosa (AN) binge purge subtype and is exhibited for both alcohol and illicit drug disorders.^1^–^5^ Findings from the few studies investigating chronology of EDs and substance use disorders suggest a bidirectional association. Cross-sectional studies reveal somewhat similar rates of women reporting onset of a substance use disorder (SUD) to precede an ED and vice versa.^6^,^7^ Moreover, in a 9-year longitudinal examination, 18% of women with AN and 30% of women with BN were diagnosed with a drug use disorder for the first time over the course of the study, suggesting that the risk for a drug use disorder continues over time in women with EDs.^8^ Similar ongoing risk has been found for alcohol disorders.^9^

In addition to illicit drug and alcohol use disorders, regular nicotine use is also frequent in women with an ED. A higher proportion of women with BN are regular smokers compared to controls or to women with AN.^5^,^10^-^12^ This association may arise because of the commonly held belief that nicotine use can aid in weight loss. Therefore, women's concerns about body weight and shape, which are particularly salient for women with an ED, may increase their risk for cigarette use.^5^

It remains unclear why EDs and SUDs frequently coexist; these associations are complex and likely have several biological and psychosocial influences. For example, when women begin to remit from an ED, they may substitute substances (i.e., dramatic increase in alcohol consumption) for the ED symptoms (i.e., binge eating) or vice versa. In addition, a common familial diathesis has been proposed. Several family studies show increased rates of SUDs in relatives of women with BN.^1^,^2^,^13^ However, several reports also suggest that these two disorders are transmitted independently.^3^,^14^–^16^ In contrast to these latter family studies, twin studies often show a familial relationship between EDs and SUDs.

Research has shown evidence for shared genetic influences between BN and illicit drug abuse/dependence.^6^ Authors reported that 83% of the phenotypic correlation between BN and illicit drug abuse/dependence was accounted for by genetic factors. However, a report examining the overlap between BN and alcohol use disorders demonstrated that the two disorders load on distinct genetic factors.^14^ Examining the genetic covariance between specific ED symptoms and substance use also reveals common genetic factors. For example, shared genetic factors were shown to account for a portion of the covariance between weight preoccupation and binge eating and alcohol use in both males and females.^17^ Thus, it may be important to examine specific ED symptoms and their relation to SUDs rather than focusing on specific diagnostic categories (AN vs. BN). This is an important consideration as recent work has emphasized the importance of examining specific symptoms of an ED noting that, “A DSM-IV diagnostic category … might actually represent an occasionally co-occurring yet etiologically diverse mixture of genetically and environmentally influenced symptoms …” (p. 191).^18^

Previous work that has examined associations between ED symptoms and SUDs has been phenotypic in nature and shows that, in general, the more severe the ED symptoms, the greater the number of substance classes used.^12^,^19^,^20^ Independent of diagnostic category, specific phenotypic associations have been shown between caloric restriction and amphetamine use and binge eating and tranquilizer use.^20^ Severe bingeing is also consistently associated with alcohol use,^19^,^21^,^22^ whereas purging behaviors have been shown to predict the use of a multitude of substances including alcohol, cocaine, cigarettes, stimulants, and amphetamines.^9^,^19-21^,^23^

This study aimed to extend previous research in this area in several ways. First, we examined the phenotypic associations between ED diagnoses (AN and BN) as well as ED symptoms. It was specifically hypothesized that significant associations would be found between binge eating and an alcohol use disorder, purging behaviors and an alcohol use disorder, illicit drug use disorder, and regular smoking and between body image and an alcohol use disorder due to previous associations found between these variables.^9^,^21^ Second, we also examined the chronology of comorbid EDs and SUDs. Finally, follow-up twin analyses were conducted between ED symptomatology and SUDs to examine for genetic covariance. We hypothesized that ED symptomatology and SUDs would show a moderate amount of genetic covariance. This report is one of the first to integrate all the above-mentioned aspects into one report using a population-based sample.

This report also adds to previous research examining similar types of associations using the Virginia Adult Twin Study of Psychiatric and substance use disorders in several ways. First, while the association between BN and illicit drug and alcohol disorders has been examined within this sample, our previous reports have only examined associations at the diagnostic level and have not examined relations between BN (at the diagnostic or symptom-level) and smoking or caffeine disorders. Second, our previous reports have neglected to include AN. Third, we examine ED diagnoses in two distinct ways (absence or presence of diagnosis and symptom count). Last, we include a detailed examination of the prevalence of comorbid ED symptomatology and SUDs including an examination of self-reported chronology of symptoms.

## Method

### Participants

Participants are from the Virginia Adult Twin Study of Psychiatric and Substance Use Disorders (VATSPSUD^24^). This is a population-based longitudinal study of adult Caucasian twins sampled from the Virginia Twin Registry (VTR, now part of the Mid-Atlantic Twin Registry). Female–female twin pairs who were born between 1934 and 1968 were targeted for the VATSPSUD. This study includes 1,206 monozygotic or identical twins (MZ) and 877 dizygotic or fraternal (DZ) female twins. Zygosity was determined by a computer algorithm of standard questionnaire responses. This method was validated by DNA testing and found to be >95% accurate.^25^

### Measures

Lifetime psychiatric and substance abuse history was assessed with an adapted version of the Structured Clinical Interview for DSM-III-R (SCID^26^). Information was drawn from interview Waves 1, 3, and 4. Participation rates at each wave were 92%, 87.8% and 84.5%, respectively.

### Lifetime SUDs

Lifetime SUDs, assessed at Wave 4 interviews, included alcohol, caffeine, cannabis, sedative, stimulant, cocaine, opiate, and hallucinogen abuse/dependence. A combined variable of abuse/dependence diagnoses was created for each of the aforementioned substances. Similarly, a variable was created to indicate whether the participant has a lifetime history of any illicit drug use disorder (including cannabis, sedative, stimulant, cocaine, opiate, and hallucinogen abuse/dependence). This was done to decrease the number of possible analyses and to increase power due to the low prevalence of illicit drug use disorders. A regular smoking variable was also included, which indicates whether the participant ever engaged in an average of at least seven episodes of smoking per month.

### Eating Disorders and Symptoms

Eating disorder (ED) symptoms were examined with the interview questions used to assess AN and BN diagnoses. Not all participants interviewed were asked the ED symptomatology questions due to use of “entry” questions. If participants did not endorse the entry questions, then all subsequent ED questions were skipped. The entry question to the BN section asked: “have you ever in your life had eating binges during which you ate a lot of food in a short period of time?” Thirty-seven percent (*n* = 718) of women responded yes to the entry question and endorsed a history of binge eating. The AN entry question asked: “have you ever had a time in your life where you weighed much less than other people thought you ought to weigh?” Thirty-six percent (*n* = 780) of women endorsed the AN entry question.

### Bulimia Nervosa

Lifetime history of bulimia nervosa (BN) was assessed at both Waves 1 and 3 interviews, and a broadly defined definition was used. Specifically, the DSM-III-R “D” criterion that bingeing and purging must occur for twice a week for 3 months was omitted, and a broader definition for concern about body weight and shape was used. This ranged from “more concerned than others about body weight and shape” to “a little bit more concerned about body weight and shape than others.” This broad definition of BN has been used previously^6^,^27^ and has been shown to be reliable.^28^ Participants were classified as having a history of BN if they qualified for a diagnosis at interview Waves 1 or 3. Our rationale was that discrepancies are more likely to be due to true change (development of problems between waves) or false negatives (underreporting at one interview) than to false positives (overreporting at one interview).

We also created a BN symptom count variable, which included the following symptoms: binge eating (assessed by the entry question), purging, which could include self-induced vomiting, laxatives, water pills, exercise, fasting, or strict dieting (a positive score was given for each purging behavior identified), feelings of loss of control during the binge, amount of food eaten during the binge, concern about weight, and shape (i.e., body image disturbance), and whether episodes of binge eating and vomiting (as the purging behavior) occurred at the same time. Symptom count information was obtained from Wave 3 interviews only due to slight differences between Waves 1 and 3 questions. For example, Wave 3 questions have more response/frequency options for the questions and include questions Wave 1 does not (i.e., amount eaten during the binge). Information from all participants was included in the symptom count variable, such that individuals not endorsing the entry item were coded as zero.

All variables were binary (yes/no) except for loss of control during the binge, amount eaten during the binge, and concern about weight and shape. For loss of control options included: not at all out of control (0), a little out of control (1), somewhat out of control (2), and completely out of control (3). Amount eaten during the binge included a small amount other's would not regard as unusual (0), a large amount others would regard as unusual (1), and a very large amount others would definitely regard as unusual (2). Concern about weight and shape included same level of concern as others (0), a little bit more concerned than others (1), somewhat more concerned than others (2), and a lot more concerned than most girls of the same age (3). All aforementioned variable responses were added together for each participant to obtain a BN symptom count, which was used in the twin analyses. The symptom count variable ranged from 0 to 11 with a mean of 1.27 (sd = 2.5).

### Anorexia Nervosa

Lifetime anorexia nervosa (AN) was obtained from Wave 1 interviews. Again, a broad definition was created due to the low prevalence of AN. Participants were considered to have a lifetime history of AN if one of the following definitions were met: (a) strict DSM-III-R criteria were met; (b) meets DSM-III-R criteria dropping criterion “D” (amenorrhea); and (c) meets DSM-III-R criteria dropping criterion “C” (feeling fat when emaciated). These definitions have been used previously within the VTR,^29^,^30^ and an etiologic continuity between these definitions was shown.^29^ Because of the low prevalence of AN in the general population and small number of concordant twin pairs in this sample, AN was not included within the twin model-fitting analyses.^29^ Because of these issues, our sample would likely not have the power to reliably detect genetic and environmental effects.

### Statistical Procedures

#### Regression Analyses

Logistic regressions were conducted using generalized estimating equation modeling (GEE^31^) as implemented within the PROC GENMOD procedure in SAS version 9.2^32^ to examine for significant associations between ED symptoms and SUDs. Using this procedure, the robust standard error is invoked, which adjusts betas and standard errors for the relatedness of twins in a pair. Members of the twin pair are identified (or clustered) within these models by a “family number” variable that is shared by both members of the pair. Broad AN and BN diagnoses were included in the models as covariates for their respective symptoms to ensure the symptoms examined have a significant relation independent of diagnosis. A more conservative *p*-value of .01 was used to assess significance for analyses because of the large number of analyses conducted.

#### Twin Analyses

For these analyses, a BN symptom count variable (now referred to as BNSC) was created (described earlier), which indicates how many symptoms of BN the participant responded to Yes. AN was not included in the twin model-fitting analyses. The symptom count variable was created for several reasons: first, to decrease the number of possible analyses conducted between ED symptoms and SUDs. This decreases the number of analyses, because it would be possible to conduct these analyses with each ED symptom. Second, this was done to increase power due to the lower prevalence of EDs in the general population. Third, researchers have now begun to highlight the importance of assessing EDs at the symptom level.^18^ Most extant research regarding genetic and environmental influences on EDs uses binary items, and participants are coded as meeting criteria for a disorder (1) or not (0). However, using a threshold imposed on a symptom count variable tells us nothing about (i) disorder liability in those who are subthreshold nor about (ii) severity among those who are above threshold. There is therefore a loss of information among both affected and unaffected individuals. To overcome this problem, we have chosen to use a symptom count variable to maximize the amount of information and power in twin-modeling analyses.

**FIGURE 1:**
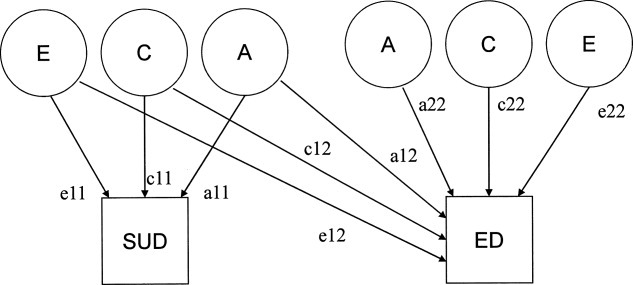
Bivariate Cholesky decomposition. *Notes:* ED, eating disorder symptom; SUD, substance use disorder; A, additive genetic; C, shared environment; E, unique environment; a11, genetic path for ED; c11, shared environmental path for ED; e11, unique environmental path for ED; a12, genetic covariance between ED and SUD; c12, shared environmental covariance between ED and SUD; e12, unique environmental covariance between ED and SUD; a22, genetic path unique to SUD; c22, shared environmental path unique to SUD; e22, unique environmental path unique to SUD.

Bivariate Cholesky decomposition models were used to decompose the covariance between BNSC and SUDs into genetic and environmental components (see Fig. 1). This model estimates regressions on standardized latent additive genetic (A), shared environmental (C), and unique environmental (E; includes measurement error) variance components. Squared, these regression coefficients provide the amount of genetic (*a*^2^), shared environmental (*c*^2^), and unique environmental (*e*^2^) variance accounted for in a trait. The Cholesky model also yields estimates of genetic and environmental contributions to the covariance between traits. These include the genetic (*r*_*a*_), shared (*r*_*c*_), and unique environmental (*r*_*e*_) correlations between the phenotypes. In the present application, these are the genetic and environmental correlations between risk factors responsible for ED symptom count and the specific SUD examined. For example, an *r*_*a*_ estimated at unity indicates that the same genetic risk factors contribute to risk for the ED symptom and SUD under investigation.

The fit of the full ACE model was compared to submodels, including models dropping C (AE model), then A (CE model), as well as A and C together (E only model). Model comparisons are conducted by taking the difference in twice the negative log-likelihood of the models, which, given certain regularity conditions, is distributed as a chi-square. A significant (*p* < .05) change in chi-square indicates that dropping the parameter significantly worsens the fit of the model. Models with fewer parameters are preferable if they do not result in a significantly worse fit. An alternative comparison method, referred to as Akaike's information criterion (AIC^33^), was used as well. AIC is calculated as χ^2^ − 2 df, which indicates the best possible balance between parsimony and explanatory power. Lower AIC values indicate better fit. The best-fitting and most parsimonious model from these analyses was retained. However, despite the fact we have a fairly large sample size, both in general and for a twin study, our sample is not large enough to reject the AE model if the true model is the CE model^34^; therefore, the discussion of the twin model-fitting results will focus on the full ACE model. The best-fitting model results will be provided for comparison to previous VATSPSUD studies.

Results of GEE analyses were used as a guide for twin analyses. Bivariate analyses were conducted between BNSC and only those SUDs that showed at least one significant association with a related ED symptom examined in GEE analyses. Analyses were conducted using an ordinal, raw data approach in the statistical package Mx,^35^ which allows data from both complete and incomplete twin pairs to be used.

## Results

### Demographics

The average ages of participants at interview Waves 1, 3, and 4 were 30.00 (sd = 7.5), 35.12 (sd = 7.5), and 37.70 (sd = 7.5), respectively. Frequency of SUDs is shown in **Table** 1. Regular smoking and a caffeine disorder were the most prevalent substances in the sample. Approximately 5% of women qualified for a BN diagnosis (*n* = 118), while 3% (*n*= 58) qualified for AN. Additionally, we assessed those women in our sample who qualified for both AN and BN (AN+BN). Six women at Wave 1 and fourteen women at Wave 3 qualified for AN + BN. Examining age of onset of those women who had a diagnosis of AN at Wave 1 and a diagnosis of BN at Wave 3 (but not Wave 1) shows that a majority (*n* = 6) reported that episodes of binge eating began 3–16 years after their age of lowest weight. This suggests to us that these women may have “progressed” from AN to BN as it is typically found that a majority of women with AN will often later develop BN after years of starvation. Therefore, AN + BN women were counted in both the AN category and the BN category.

**TABLE 1:** Frequency of substance disorders and of women with anorexia nervosa or bulimia nervosa and a substance disorder

	Prevalence of substance disorders		Anorexia nervosa and comorbid substance disorder			Bulimia nervosa and comorbid substance disorder		
	%	*N*	%	*N*	OR (95% CI)	%	*N*	OR (95% CI)
Any illicit drug disorder^a^	8.14	177	17.20	10	2.00 (0.85–4.40)	18.60	22^b^	2.39^c^ (1.40–4.10)
Alcohol	14.00	235	22.40^b^	13	2.30^c^ (1.20–4.50)	24.00	28^b^	2.83^c^ (1.79–4.50)
Caffeine	23.00	382	26.00	15	1.52 (0.78–3.00)	23.00	27	1.40 (1.20–2.23)
Regular smoker	43.00	740	52.00^b^	30	2.03 (1.10–3.70)	45.00^d^	53	1.60 (1.10–2.50)

*Note:* %, percentage of women; *N*, number of women; OR, odds ratio from Generalized Estimating Equation Modeling; CI 95%, confidence interval for odds ratio.

a All illicit drug abuse/dependence diagnoses combined into one variable for ease of comparison across eating pathology.

b Significant chi-square difference between women positive for AN or BN diagnosis and women scoring zero at *p* < .01.

c GEE analysis significant at *p* < .01.

d Significant chi-square difference between women positive for AN or BN diagnosis and women scoring zero at *p* < .05.

Average age of onset for AN and BN was also examined. For AN, we examined the self-reported age of lowest weight of those individuals who qualified for an AN diagnosis. Mean age of onset for AN was 18.80 (sd = 4.8), which is slightly higher than the age of onset in the general population. This higher than average age of onset is likely due to using the participant's age at lowest weight as age of onset, and it is probable that onset of AN would occur well before the participant reached their lowest weight.

Age of onset for BN was examined at interview Waves 1 and 3. For Wave 1, the question “how old were you when you first began to have these symptoms” was asked at the end of the BN section and examined in those women who qualified for BN. Reported mean age of onset was 18.60 (sd = 5.3). Age of onset at Wave 3 was obtained in two ways: first, by inquiring the age at which binge eating began and, second, by inquiring at what age self-induced vomiting behaviors began in those women with BN. Average age of onset of binge eating for those women with BN was 20.0 (sd = 7.4), while the average age of onset of self-induced vomiting was 21.50 (sd = 6.4).

### Prevalence of Comorbid Eating Pathology and SUD

#### ED Diagnoses and SUDs

Frequency of SUDs was examined in women with AN or BN. Chi-squares revealed that women with BN were more likely to have an illicit drug use disorder (1, *N* = 1715) = 12.32, *p* < .01, alcohol disorder (1, *N* = 1719) = 20.70, *p* < .01, and be regular smokers (1, *N* = 1720) = 5.20, *p* < .05, compared to those without a BN diagnosis. Women with AN were more likely to have an alcohol disorder (1, *N* = 1719) = 6.63, *p* < .01 and be regular smokers (1, *N* = 1720) = 6.05, *p* < .01, compared to women without an AN diagnosis. The chi-square difference between rates of an illicit drug use disorder in women with and without an AN diagnosis approached significance (1, *N* = 1715) = 3.40, *p* = .06.

As can be seen from **Table** 1, the prevalence of SUDs in women with AN or BN was quite similar. The largest differences were exhibited for a caffeine disorder and regular smoking, both of which were more prevalent in women with AN. Twenty-six percent of women with AN had a caffeine disorder, and 52% were regular smokers compared to 23 and 45% of women with BN, respectively. However, chi-squares revealed no significant differences between women with AN and BN and any SUD prevalence rates (*p*'s > .05).

We also examined the prevalence of the specific illicit drug disorders that comprised our “any illicit drug disorder” variable. Cannabis (13%, *n* = 15), stimulant (9.3%, *n* = 11), and cocaine abuse/dependence (9.3%, *n* = 11) were the most prevalent illicit SUDs within BN. Similarly, cannabis (13.8%, *n*= 8), stimulant (13.8%, *n* = 8), and cocaine abuse/dependence (12.1%, *n* = 7) were also the most prevalent illicit SUDs reported in women with AN.

The chronology of the onset of AN, BN, and SUDs revealed a bidirectional relationship (**Table** 2). Only those women having age of onset information for both disorders were included. In general, women with AN reported that their SUD symptoms (positive report for any symptoms of abuse/dependence) preceded their symptoms of AN (based on age at lowest weight in those women with AN), whereas women with BN reported their symptoms of BN (based on age binge eating began in those women with BN) preceded their SUD symptoms. The only exceptions to this were for BN and regular smoking where more women reported regular smoking (for at least a month) to precede binge eating and for AN and an alcohol use disorder where more women reported age of lowest weight to occur before the alcohol disorder. Age of onsets for an illicit drug use disorder and a caffeine disorder was determined in a similar fashion to the previous SUDs mentioned. Age of onset for an illicit drug use disorder diagnosis was determined by the minimum possible age for an abuse/dependence diagnosis for any of the illicit drugs, whereas age of onset for a caffeine disorder was determined by the age of heaviest caffeine use in those women with a caffeine disorder diagnosis.

**TABLE 2:** Chronology of comorbid eating disorder and substance use disorder onset

Substance disorder	BN + SUD BN precedes	BN + SUD SUD precedes	BN + SUD same age of onset	AN + SUD AN precedes	AN + SUD SUD precedes	AN + SUD Same age of onset
Illicit drug disorder	57% (*n* =12)	39% (*n* = 8)	5% (*n* = 1)	33% (*n* = 3)	67% (*n* = 6)	—
Alcohol disorder	70% (*n* = 14)	15% (*n* = 3)	15% (*n* = 3)	67% (*n* = 6)	33% (*n* = 3)	—
Caffeine disorder	70% (*n* = 9)	23% (*n* = 3)	8% (*n* = 1)	50% (*n* = 3)	50% (*n* = 3)	—
Regular smoking	52% (*n* = 16)	39% (*n* = 12)	10% (*n* = 3)	36% (*n* = 9)	60% (*n* = 15)	4% (*n* = 1)

*Note:* Only those women with age of onset information for both disorders were included. BN + SUD, bulimia nervosa and substance use disorder; BN precedes, bulimia nervosa age of onset precedes substance disorder onset; SUD precedes, substance use disorder age of onset precedes eating disorder onset; same age of onset, reported age of onset for eating disorder and substance use disorder the same; AN + SUD, anorexia nervosa and substance use disorder.

#### ED Symptomatology

The prevalence of SUDs within women positive for specific ED symptoms was also examined, and results are provided in **Table** 3. In general, regular smoking and a caffeine disorder were again the most prevalent SUDs in women positive for an ED symptom. All SUDs examined were most prevalent among women reporting lifetime purging, except for a caffeine disorder, which was slightly more prevalent in women who are concerned about their weight and shape. A significant difference was shown between those women concerned about their weight and shape and those who are not in regard to a caffeine disorder (1, *N* = 657) = 12.00, *p* < .01. Additionally, there was a significant chi-square difference between those women with a history of purging and those without for any illicit drug use disorder (1, *N* = 1707) = 30.82, *p* < .01, an alcohol disorder (1, *N* = 1711) = 27.00, *p* < .01, and regular smoking (1, *N* = 1712) = 27.70, *p* < .01. Similarly, of those symptoms related to AN, believing one is overweight when others believed that the participant was underweight had the highest prevalence of all the SUDs examined. However, differences were not significant (**Table** 3).

**TABLE 3:** Frequency of substance disorders within women positive for eating disorder symptomatology

	Illicit drug disorder^a^			Alcohol disorder			Caffeine disorder			Regular smoking		
Eating disorder symptom	%	*N*	OR (95% CI)	%	*N*	OR (95% CI)	%	*N*	OR (95% CI)	%	*N*	OR (95% CI)
Symptoms related to BN												
Binge eat	14.20^b^	102	2.03^c^ (1.46–2.83)	17.00^b^	121	2.01^c^ (1.50–2.72)	23.00^b^	165	1.63^c^ (1.26–2.10)	42.80^b^	307	1.65^c^ (1.32–2.08)
Purging behaviors	17.30^b^	51	2.61^c^ (1.63–4.20)	20.10^b^	59	2.01^c^ (1.26–3.32)	22.80	67	1.25 (0.84–1.90)	48.60^b^	143	2.46^c^ (0.70–3.56)
Concern about weight/shape Symptoms related to AN	14.60^b^	60	1.80^c^ (1.20–2.60)	17.80^b^	73	1.80^c^ (1.26–2.60)	24.00^b^	98	1.63^c^ (1.20–2.24)	45.00^b^	184	1.80^c^ (1.34–2.36)
Believe overweight	15.00	18	1.60 (0.85–3.00)	18.30	22	1.60 (0.80–3.00)	28.00	33	2.01^c^ (1.20–3.50)	44.20	53	1.30 (0.79–2.16)
Fear of gaining weight	14.70	26	1.73 (1.00–3.03)	16.40	29	1.43 (0.82–2.51)	26.00	46	1.93^c^ (1.22–3.10)	42.40	75	1.31 (0.85–2.03)
Amenorrhea	12.20	14	0.94 (0.47–1.90)	12.20	14	0.79 (0.40–1.54)	25.20	29	1.26 (0.77–2.05)	37.40	43	0.73 (0.46–1.20)
85% below ideal weight	13.20	57	1.35 (0.80–2.30)	13.00	55	0.80 (0.50–1.28)	19.00	82	0.67 (0.45–1.00)	39.00	168	0.74 (0.52–1.07)

*Note:* BN, bulimia nervosa; AN, anorexia nervosa; %, percentage of women positive for specific eating disorder symptom who also qualify for a substance disorder diagnosis; *N*, number of women positive for specific eating disorder symptom who also qualify for substance disorder diagnosis; OR, odds ratio from Generalized Estimating Equation (GEE) Modeling; CI, 95% confidence interval for odds ratio.

a All illicit drug abuse/dependence diagnoses combined into one variable for ease of comparison across eating pathology.

b Significant chi-square difference between women positive for symptom and women scoring zero at *p* < .01.

c GEE analysis significant at *p* < .01.

### GEE Analyses

As can be seen from **Table** 4, very few AN-related symptoms were significantly associated with the SUDs. However, believing one is overweight when underweight and fear of gaining weight were significantly associated with a caffeine use disorder. Having this disturbance in body image increased the chances of a caffeine disorder diagnosis by ∼2. AN diagnosis was significantly associated with an alcohol use disorder (**Table** 1). Similar to body image disturbances, there was a twofold increase of an alcohol use disorder diagnosis in women with AN.

**TABLE 4:** Model-fitting results from bivariate Cholesky models

	*r*_*a*_		*r*_*c*_		*r*_*e*_		Δχ^2^; (*p)*	AIC
Model	Estimate	95% CI	Estimate	95% CI	Estimate	95% CI	Estimate	95% CI
Alcohol disorder and BNSC								
ACE model	.53	−0.02; 1.00	.32	−1.00;1.00	−.03	−0.24; 0.18	—	−1783.44
AE model^a^	.53	0.30; 0.80	—	—	−.03	−0.24; 0.18	0	−1789.44
Caffeine disorder and BNSC								
ACE model	.35	−1.00; 1.00	1.00	−1.00;1.00	.02	−0.15; 0.20	—	−1310.34
AE model^a^	.35	0.03; 0.73	—	—	.02	−0.15; 0.20	0	−1316.35
Illicit drug disorder and BNSC								
ACE model	.37	−0.17; 1.00	0.83	−1.00;1.00	.23	−0.01; 0.46	—	−1942.22
AE model^a^	.37	0.15; 0.58	—	—	.23	−0.01; 0.46	0	−1948.22
Regular smoking and BNSC								
ACE model	.25	−0.30; 1.00	1.00	−1.00;1.00	.05	−0.18; 0.28	—	−1022.23
AE model^a^	.35	0.20; 0.51	—	—	.04	−0.20; 0.27	.67; (.88)	−1299.75

*Note: r_a_*, genetic correlation; *r*_*c*_, shared environmental correlation; *r*_*e*_, unique environmental correlation. Δχ^2^, change in chi-square from full model. *p*, *p*-value associated with change in chi-square; AIC, Akaike's information criterion; 95% CI, 95% confidence interval; BNSC, bulimia symptom count.

a Best-fitting model according to AIC.

Unlike AN symptomatology, several symptoms related to BN were significantly associated with the SUDs (**Table** 4). Binge eating and body image were significantly associated with all the SUDs examined. Results showed a twofold increase in these SUDs in women who binge eat or have a negative body image. Purging behaviors were significantly associated with alcohol and illicit drug disorders as well as regular smoking. Women who engage in purging behaviors were two to three times more likely to have an alcohol or illicit drug disorder or to smoke regularly. Finally, BN diagnosis was significantly related to an alcohol or illicit drug use disorder increasing the likelihood of having one of these SUDs by ∼3 (**Table** 1).

### Biometric Twin Analyses

#### Initial Analyses

Before conducting our bivariate analyses, we performed univariate analyses to examine the genetic and environmental influences on BNSC and the SUDs. We examined the full ACE model and compared the fit to the AE, CE, and E models previously described. The AE model was the best-fitting model for BNSC. Results suggest that genetic factors account for 42% (95% CI: 7–53%) and unique environmental factors account for 58% (95% CI: 47–70%) of the variance.

For the SUDs, we only examined the full ACE model to decrease the number of results reported as this was not a main goal of our analyses, and most SUDs examined within this report have been examined in previous VATSPSUD works. All substances revealed very little shared environmental effects. Regular smoking showed the largest shared environmental effects at 5% (95% CI: 0–34%), with 81% (95% CI: 50–91%) genetic, and 14% (95% CI: 10–21%) unique environmental variance. Illicit drug use disorders had the second largest heritability estimated at 68% (95% CI: 32–80%), with the rest of the variance attributed to unique environment, 32% (95% CI: 20–47%). Heritability estimates for an alcohol use disorder and a caffeine disorder were 53% (95% CI: 7–68%) and 27% (95% CI: 0–42%), respectively. Again, the rest of the phenotypic variance was attributed to unique environmental factors for both an alcohol (47%; 95% CI: 32–64%) and a caffeine disorder (73%; 95% CI: 58–90%).

#### Bivariate Twin Analyses

Because at least one BN-related symptom was significantly associated with the SUDs examined in the GEE analyses, bivariate twin analyses were conducted with BNSC and all available SUDs. As shown in **Table 5**, the strongest genetic association was between BNSC and an alcohol use disorder with an estimated genetic correlation of +.53 (95% CI: −0.02; +1.00). The smallest genetic association was between BNSC and regular smoking with an estimated genetic correlation of +.25 (95% CI: −0.30; +1.00). However, confidence intervals for the shared environmental correlations range from −1.00 to +1.00 for all analyses as well as the confidence interval for the genetic correlation between BNSC and a caffeine disorder. Confidence intervals that include zero suggest nonsignificance. Therefore, *r*_*a*_ and *r*_*c*_ may be nonsignificant individually. However, because the unique environmental correlations do not include ±1.00, familial factors are important for the overlap between BNSC and the SUDs. Our data appear to be insufficient to determine whether the origin of familial coaggreation is genetic, shared environmental, or both.

## Discussion

The purpose of this study was to examine the association between ED diagnoses, their symptomatology, and SUDs. Several important findings emerged. First, consistent with the previous literature,^2^ ED diagnoses were significantly related to SUDs. Specifically, women with AN had a twofold increased risk of having an alcohol use disorder and being regular smokers. Women with BN were two to three times more likely to have an alcohol or illicit drug use disorder and be regular smokers. However, in contrast to previous reports, which generally find SUDs to be significantly more common in women with BN compared to AN,^2^ our results showed no significant differences between the prevalence rates of SUDs in women with AN and BN. Additionally, while results were not statistically significant, a caffeine disorder and regular smoking were more prevalent in women with AN compared to BN. These differences could be due to the fact that our sample is community-based as opposed to clinical. The nonsignificant increased rates of a caffeine disorder and regular smoking in women with AN also could be due to the common belief that both these substances increase metabolism. The association between EDs and a caffeine disorder is also not well studied.

Second, results revealed that, in general, BN manifests before a SUD. Of women with age of onset information for both disorders, most reported binge eating preceded symptoms of a SUD. This may be occurring because of several possibilities. First, these women may be beginning to remit from BN and be substituting substances for the ED symptoms. Second, the women may be using substances to dampen bulimic urges or negative affect. Third, both disorders may be caused by a common factor, whereas BN symptoms simply have an earlier onset. In contrast, more women with AN reported SUD symptoms preceded their age of lowest weight. The only exception to this was AN and an alcohol use disorder. It could be hypothesized that women with AN begin experimenting with substances before the onset of AN (or before a diagnosis can be made) in an effort to lose weight. Additional possibilities for this pattern could be the age of onset information used for AN in this report was the age at lowest weight. Second, AN diagnosis requires a specific amount of weight to be lost before a diagnosis can be made; therefore, this finding may be a reflection of our data or of AN diagnostic criteria.

Third, as hypothesized, several of the ED symptoms examined were significantly associated with SUDs. For AN symptomatology, only the variables reflecting body image (fear of gaining weight and belief overweight when underweight) were significantly associated with a SUD. The presence of negative body image in women who reported having been under weight previously increased the risk for a caffeine use disorder by ∼2. A similar pattern was revealed for BN-related symptoms. Concern about weight and shape in women with a history of binge eating was significantly associated with all of the SUDs examined, again increasing risk by ∼2. It is important to note, however, that these body image symptoms are likely more common in our sample and show greater variability compared to other ED symptomatology, which may account for the large number of significant associations revealed. Finally, binge eating and purging behaviors were significantly related to a multitude of SUDs. Binge eating was associated with all SUDs, whereas purging behaviors in women with a history of binge eating showed significant associations with all expect a caffeine use disorder. These results confirm our hypotheses, based on previous reports, that binge eating, purging behaviors, and body image would be associated with an alcohol use disorder and that purging behaviors would be significantly associated with illicit drug use disorders and regular smoking. However, we did not find a significant relation between AN body image and an alcohol use disorder identified by previous research.^9^

Forth are the findings from the twin analyses. To date, few studies have examined the genetic and environmental covariance between ED symptomatology and SUDs. Moreover, a limited number of investigations have examined BN using a symptom count. The univariate BNSC genetic and environmental estimates are similar to our previous examination of broadly defined BN diagnosis in VATSPSUD.^6^

Bivariate analyses indicate a shared etiology between ED symptomatology and SUDs. Although our data may not have been sufficient to differentiate between genetic and shared environmental effects on comorbidity, our results do indicate that familial factors are important for the overlap between these two disorders, which is consistent with previous research.^6^,^17^ Because no *r*_*e*_ confidence intervals included 1.00, we know that individual-specific environmental factors are not sufficient to account for the overlap. Estimated genetic correlations from the ACE models ranged from +.25 to +.53, with the highest correlation between BNSC and an alcohol use disorder. This contrasts a previous VATSPSUD report investigating genetic covariance between alcoholism and BN diagnosis (along with four other disorders).^14^ Kendler and colleagues^14^ noted that most of the genetic factors responsible for alcoholism were disorder specific and unrelated to the factors that influence BN (as well as mood and anxiety disorders). Differences could have arisen, because the previous report used a broad definition of alcoholism, a differing definition of BN, a best-fit model, and a multivariate common factor model.

Results also indicate that the relationship between BNSC and SUDs may not be substance specific as a majority of the SUDs examined showed common familial influences with BNSC. This result makes sense in the light of substance use literature that shows genetic influences on both substance use and SUDs is not substance specific, but produces a general vulnerability to use or misuse.^36^–^38^ This common familial susceptibility to BN-related behaviors and substance misuse may simply place an individual at risk for both, whereas additional specific factors determine the exact type of substance used (e.g., availability). This may also provide limited evidence for BN as an addictive disorder. It could be that there is a general vulnerability to BN and substance misuse and that additional specific factors (e.g., personality) determine which behaviors arise.

Finally, this is one of the first studies to examine the comorbidity of ED symptomatology and a caffeine disorder. Although neither AN nor BN diagnoses were significantly associated with a caffeine disorder in GEE analyses, several ED symptoms were. Body image related to both AN and BN as well as binge eating were significantly related to a caffeine disorder, corroborating research showing a correlation between binge frequency and caffeine use.^10^

There are limitations to this study that warrant discussion. First, and perhaps most noteworthy, is the way our ED symptom variables were created, specifically the use of entry questions. Although the use of entry questions is common practice in large scale, interview-based studies to ease the burden of participants; this can be problematic when examining symptomatology. Although the entry questions are part of the diagnostic criteria for AN or BN, we still are not able to obtain “true” symptom counts as it is possible to have participants whom purge but not binge eat, for example.

Second is our insufficient power to differentiate between genetic and shared environmental effects. Although our sample size is large in terms of both general population and twin samples, we still did not have the power to reject the AE model if the CE model was the true, best-fitting model. However, for a small effect size, an exceptionally and possibly unreasonably large sample of twin pairs would be needed. Despite the inability to differentiate between common genetic and shared environmental effects, results still provide clear evidence for the importance of familial factors impacting the comorbity between BNSC and SUDs. Third is the retrospective nature of the data. This method can have an impact on the reliability and validity of the twins' reports. However, BN diagnosis within our Wave 3 sample has been shown to be reliable.^28^

A fourth potential limitation is merging substance abuse and dependence into a combined variable. Importantly, however, examinations within our own data have never produced results, suggesting distinct genetic risk factors for alcohol abuse versus dependence^25^,^39^ and other substances.^40^ Therefore, collapsing categories would not have a significant impact on results. Other limitations include the fact that women who were born between 1934 and 1968 were ascertained for the VATSPSUD, so there could be age or cohort effects on results and the use of broader definitions of AN and BN.

Despite the limitations of this report, it also has several strengths including the use of ED diagnoses, symptoms, and a BN symptom count variable. As discussed, research is emphasizing the importance of examining ED symptoms as opposed to diagnoses, which might only represent occasionally co-occurring etiological diverse symptoms.^18^ We attempted to address this issue by including symptoms rather than relying solely on diagnoses in our investigations. Second, we thoroughly assessed the temporal relationship of AN, BN, and SUDs. A limited amount of studies have examined this in both AN and BN. Third, we used a population-based sample, increasing the generaliability of our results.

Current findings have important implications for future research as well as clinical work. Further investigations regarding genetic and environmental influences on associations among specific ED symptoms and SUDs are important for treatment and prevention efforts. Moreover, women presenting with either an ED or a SUD should be assessed for symptoms of the other disorder. Importantly, women with subthreshold levels of EDs may be vulnerable to developing a SUD, as specific symptoms as well as overall diagnosis, were related to substance use. EDs continue to be among the most difficult psychiatric disorders to treat. Continued elucidation of predisposing and maintaining factors, including comorbid relationships, is essential in addressing these pernicious behaviors.

### Earn CE credit for this article!

Visit: http://www.ce-credit.com for additional information. There may be a delay in the posting of the article, so continue to check back and look for the section on Eating Disorders. Additional information about the program is available at http://www.aedweb.org
